# 

**Published:** 1889-05

**Authors:** 


☞ We have given you, Doctor, not only a handsome edition of the JOURNAL with 24 extra pages and a *souvenir* moss type engraving of our popular President—but a full report of all that was done at the big meeting at San Antonio. Every member should consider it his *bounden duty* to, at once send in his subscription, to aid and encourage, by every means in his power, a journal which is *con amore*, so devoted to the interests of the Association, and so studious of the needs of the members. It "fills a long felt want," and if it is not absolutely indispensable to members, they will find it most useful in keeping them posted on all matters of interest to the profession in the State. Don't wait, but write *now*, and if it is not convenient to send $2, say you will "remit in the fall," and it will be O. K. Sixty-four new subscribers acknowledged their allegiance in San Antonio, and thus,—tall oaks attain a maximum of altitude from diminutive beginnings, or words to that effect.

